# Supplementary material for: Dot1 regulates nucleosome dynamics by its inherent histone chaperone activity in yeast
Source: Nat Commun. 2018 Jan 16;9:240. doi: 10.1038/s41467-017-02759-8 (PMC5770421; doi:10.1038/s41467-017-02759-8)
Supplement: Supplementary file 1 — Supplementary Information [file 41467_2017_2759_MOESM1_ESM.pdf]

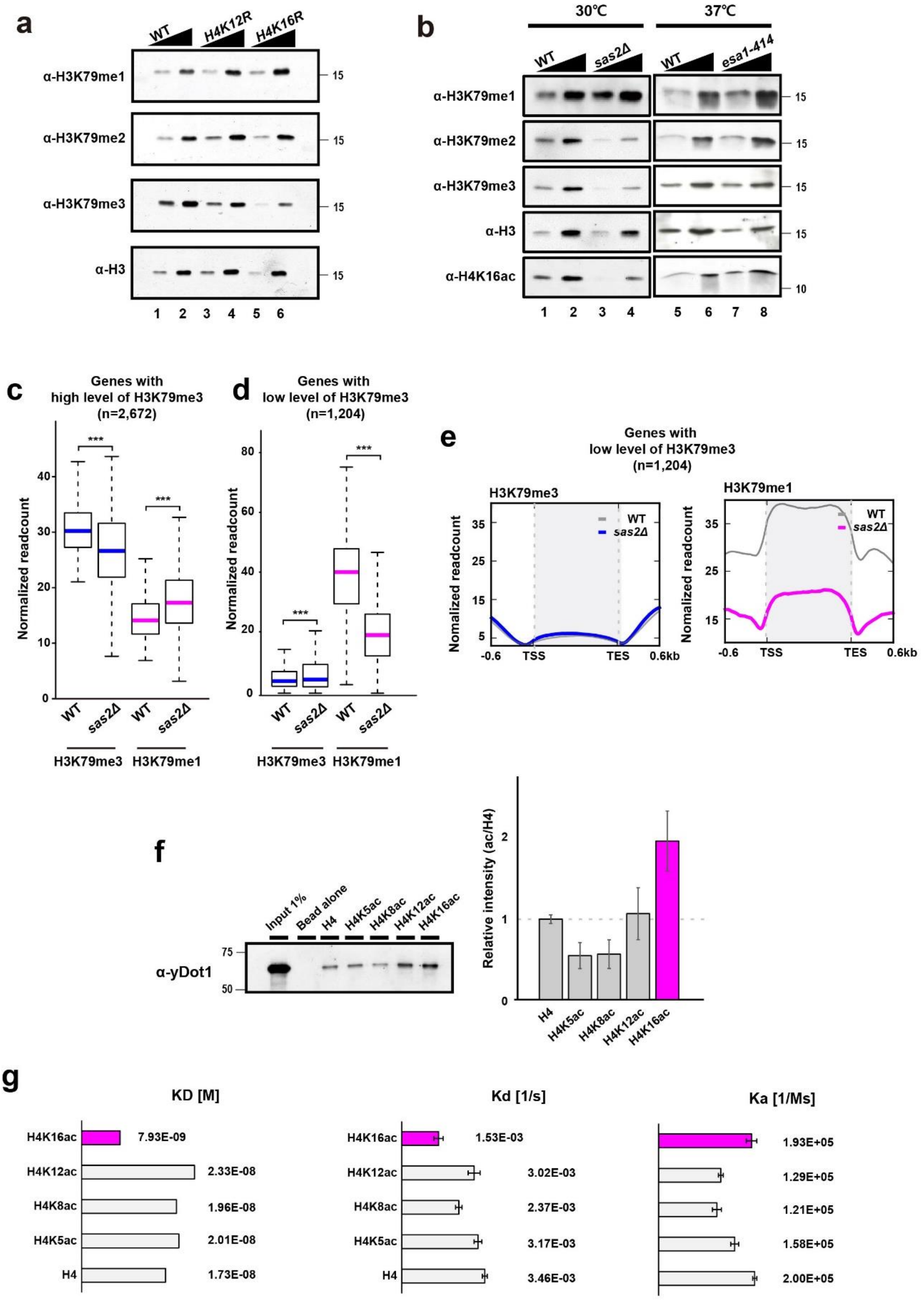
a
WT
H4K12R
H4K16R
α-H3K79me1
α-H3K79me2
α-H3K79me3
α-H3
15
1 2 3 4 5 6
b
30℃
37℃
WT
sas2Δ
WT
esa1-414
α-H3K79me1
α-H3K79me2
α-H3K79me3
α-H3
α-H4K16ac
15
10
1 2 3 4 5 6 7 8
c
Genes with
high level of H3K79me3
(n=2,672)
Normalized readcount
***
WT
sas2Δ
H3K79me3
H3K79me1
d
Genes with
low level of H3K79me3
(n=1,204)
Normalized readcount
***
WT
sas2Δ
H3K79me3
H3K79me1
e
Genes with
low level of H3K79me3
(n=1,204)
H3K79me3
H3K79me1
Nomalized readcount
WT
sas2Δ
-0.6
TSS
TES
0.6kb
f
Input 1%
Bead alone
H4
H4K5ac
H4K8ac
H4K12ac
H4K16ac
α-yDot1
75
50
Relative intensity (ac/H4)
g
KD [M]
H4K16ac 7.93E-09
H4K12ac 2.33E-08
H4K8ac 1.96E-08
H4K5ac 2.01E-08
H4 1.73E-08
Kd [1/s]
H4K16ac 1.53E-03
H4K12ac 3.02E-03
H4K8ac 2.37E-03
H4K5ac 3.17E-03
H4 3.46E-03
Ka [1/Ms]
1.93E+05
1.29E+05
1.21E+05
1.58E+05
2.00E+05

Supplementary Figure 1. Dot1 has a binding preference for Sas2-mediated H4K16ac

(a, b) Samples were collected by trichloroacetic acid (TCA) precipitation and subjected to Western blotting. (a) Plasmids encoding histone H4 with lysine-to-arginine mutations were inserted into the wzy42 strain. Wild-type (lanes 1, 2), *H4K12R* (lanes 3, 4), and *H4K16R* (lanes 5, 6) samples were loaded with a gradient of the total protein concentration. (b) Wild-type (lanes 1, 2) and *sas2Δ* (lanes 3, 4) cells were grown at 30°C, while the temperature-sensitive mutant, *esa1-414* (lanes 7, 8) and its wild-type control (lanes 5, 6) were grown at 37°C.

(c, d) Boxplot of normalized read count ChIP-seq data. H3K79me3 is indicated in blue, while H3K79me1 is shown in red. (c) H3K79me3 and H3K79me1 at transcribed regions of wild-type cells; the plot represents RPKM values calculated for genes with a high level of H3K79me3 (n=2,672). (d) H3K79me3 and H3K79me1 at transcribed regions of wild-type cells; the plot represents RPKM values calculated for genes with a low level of H3K79me3 (n=1,204). Asterisks (***) indicates p-value <0.001 (Wilcoxon-and-Mann-Whitney tests).

(e) Average plot of H3K79me3 (upper) and H3K79me1 (bottom) in wild-type and *sas2Δ* cells at genes showing a low level of H3K79me3 (n=1,204).

(f) Peptide pull-down assay indicates that binding preference of Dot1p to H4 tail which acetylated lysine peptides (H4, H4K5ac, H4K8ac, H4K12ac, H4K16ac). The bar graph represents values quantified from three experimental replications; error-bars show the s.d. for the biological replicates (right).

(g) The calculated equilibrium dissociation constant, K_D[M], dissociation constant, K_d[1/s], and association constant, K_a[1/Ms], for Dot1p at each H4 tail peptide, as determined using the BLItz system.

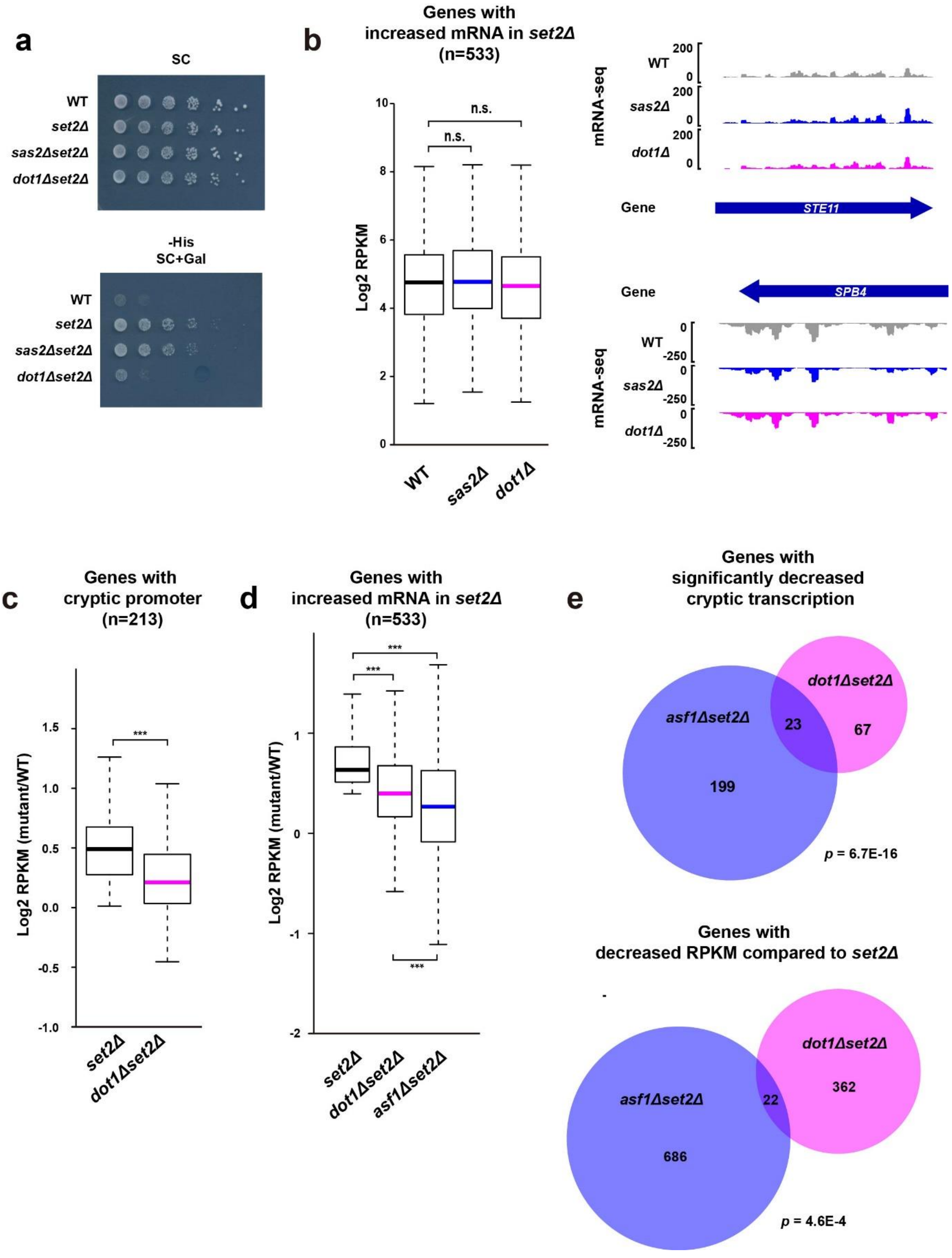
a
SC
WT
set2Δ
sas2Δset2Δ
dot1Δset2Δ
-His
SC+Gal
WT
set2Δ
sas2Δset2Δ
dot1Δset2Δ
b
Genes with
increased mRNA in set2Δ
(n=533)
n.s.
n.s.
Log2 RPKM
10
8
6
4
2
0
WT
sas2Δ
dot1Δ
mRNA-seq
WT
sas2Δ
dot1Δ
200
0
Gene
STE11
Gene
SPB4
mRNA-seq
WT
sas2Δ
dot1Δ
0
-250
c
Genes with
cryptic promoter
(n=213)
***
Log2 RPKM (mutant/WT)
1.5
1.0
0.5
0.0
-0.5
-1.0
set2Δ
dot1Δset2Δ
d
Genes with
increased mRNA in set2Δ
(n=533)
***
***
***
Log2 RPKM (mutant/WT)
1
0
-1
-2
set2Δ
dot1Δset2Δ
asf1Δset2Δ
e
Genes with
significantly decreased
cryptic transcription
asf1Δset2Δ
dot1Δset2Δ
199
23
67
p = 6.7E-16
Genes with
decreased RPKM compared to set2Δ
asf1Δset2Δ
dot1Δset2Δ
686
22
362
p = 4.6E-4

Supplementary Figure 2. Dot1p plays a role similar to that of Asf1 for cryptic transcription in Set2-Rpd3S pathway-disrupted cells

(a) Spotting assay using *pGAL1-FLO8-HIS3* reporter strains. Wild-type and mutant strains were diluted 5-fold and spotted onto synthetic complete (SC) medium supplemented with 2% glucose (upper panel) and histidine-lacking SC medium supplemented with 2% galactose (-His SC+Gal, lower panel). Plates were incubated at 30°C for 3 days.

(b) A Boxplot of the log2 mRNA-seq RPKM values of wild-type (black), *sas2Δ* (blue), and *dot1Δ* (magenta) mutants for genes that showed increased mRNA expression in *set2Δ* cells (number of genes, n=533) (left panel). mRNA-seq data in wild-type (grey), *sas2Δ* (blue), and *dot1Δ* (magenta) mutants for the cryptic loci, *STE1* and *SPB4*. The plus and minus values of the y-axis indicate level of mRNA read counts and the sense and antisense strands, respectively (left to right, +; right to left, -). The read count data were obtained from biological duplicates (right panel). The abbreviation n.s. indicates p-value>0.05 (Wilcoxon-and-Mann-Whitney tests).

(c) A Boxplot of $\log_2$ fold-change values for *set2Δ* (black) and *dot1Δset2Δ* (magenta) mutants versus wild type in the reference group of cryptic genes (n=213). Asterisks (***) indicates p-value <0.001 calculated by Wilcoxon-and-Mann-Whitney tests.

(d) A Boxplot of $\log_2$ fold-change values for *set2Δ* (black), *dot1Δ set2Δ* (magenta), and *asf1Δset2Δ* (blue) mutants versus wild type for genes sorted based on increased mRNA expression in *set2Δ* cells (n=533). Asterisks (***) indicates p-value <0.001 calculated by Wilcoxon-and-Mann-Whitney tests.

(e) Venn diagrams. In the left panel, gene groups were separated on the basis of clear mRNA down-regulation in double mutants compared to those exhibiting increased mRNA in *set2Δ* cells (n=533), as assessed using Cuffdiff ($\log_2$ fold change <-0.4; p-value <0.05). The colored circles indicate groups of genes from *dot1Δset2Δ* (magenta, n=90) or *asf1Δset2Δ* (blue, n=222) cells. The right panel shows gene groups that exhibited mRNA down-regulation in double mutants compared to *set2Δ* cells (assessed using Cuffdiff). Colored circles indicate groups of genes from *dot1Δset2Δ* (magenta, n=384) or *asf1Δset2Δ* (blue, n=708) cells. Hypergeometric *P* value is 6.7E-16 (upper panel) and 4.6E-4 (lower panel).

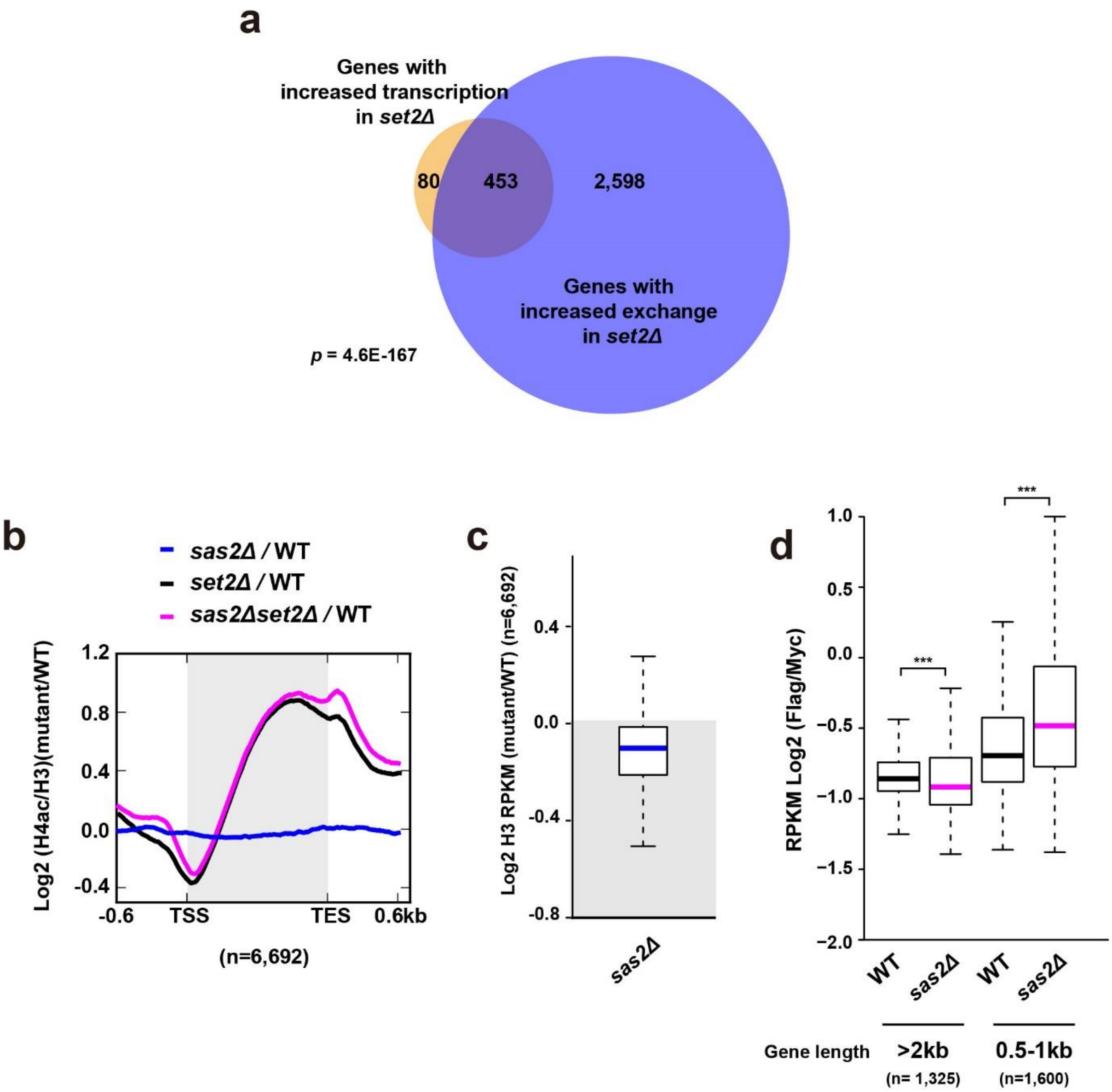

Supplementary Figure 3. Cryptic transcription overlaps with increased histone exchange in *set2Δ* cells

(a) A Venn diagram depicting the overlap between genes that show increased histone exchange across transcribed regions (blue, n=3,053) and those that show increased mRNA expression (cryptic expression) following the deletion of *SET2* (orange, n=533). Hypergeometric *P* value is 4.6E-167.

(b) The ChIP-seq average plot of H4ac normalized by H3. The values for *set2Δ* (black),

sas2Δset2Δ (magenta), and *sas2Δ* (blue) cells were calculated as the log_2 ratios of the mutant over wild type. The values of the y-axis indicate log2 fold change level of H4ac/H3 ChIP-seq in mutants over wild-type cells. The data set included all transcribed regions of yeast (number of genes, n=6,692), and were obtained from biological duplicates.

(c) Boxplot of histone H3 log_2 fold-change values obtained for *sas2Δ* (blue) at the transcribed regions of all yeast genes (number of genes, n=6,692). The y-axis indicates log_2 fold change of H3 in mutants over wild-type. The shadowed box indicates negative values, which mean decrease in mutant compared to wild-type.

(d) Histone turnover is slightly decreased at the centers of transcribed regions with long-length genes in *sas2Δ* cells. A boxplots of Flag/Myc RPKM values obtained at the centers of the transcribed regions for each gene length cluster (i.e., >2 kb and 0.5-1 kb). Asterisks (***) indicates p-value <0.001 calculated by Wilcoxon-and-Mann-Whitney tests.

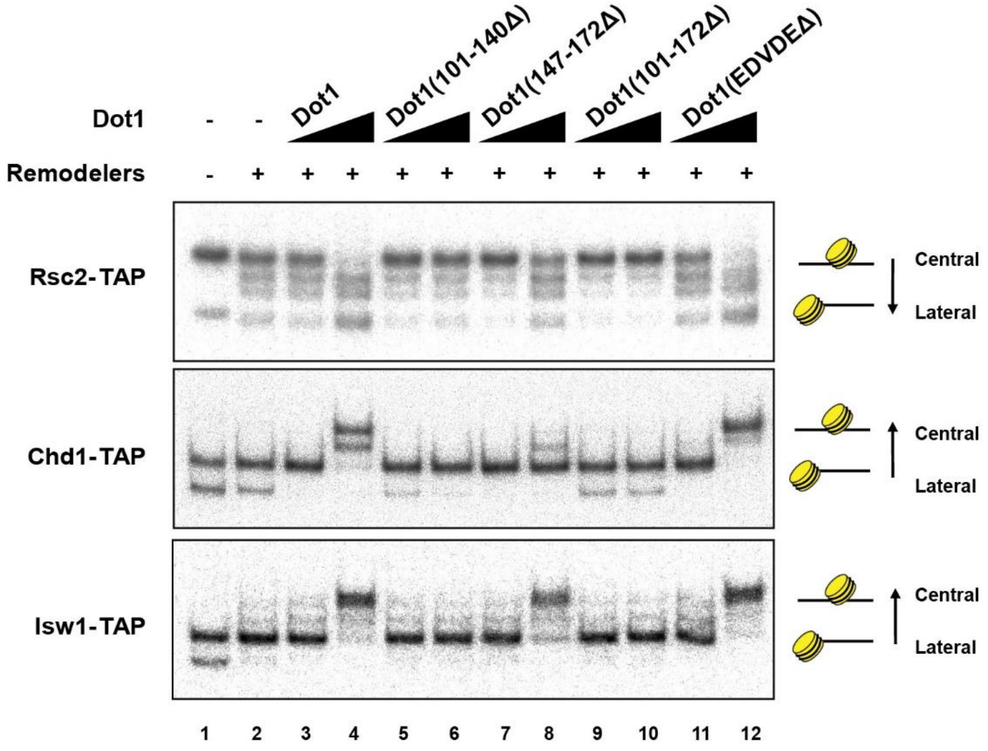

Supplementary Figure 4. Dot1 stimulates nucleosome-remodeling activity regardless of the remodeler type

Sliding assays for Rsc2p, Chd1p, and Isw1p were performed with Dot1p (lanes 3, 4), nucleosome binding-defective mutant Dot1(101-140Δ) (lanes 5, 6), DNA-binding-defective mutant Dot1(147-172Δ) (lanes 7, 8), Dot1(101-172Δ) (lanes 9, 10), and H4 tail binding-defective mutant EDVDEΔ (lanes 11, 12).

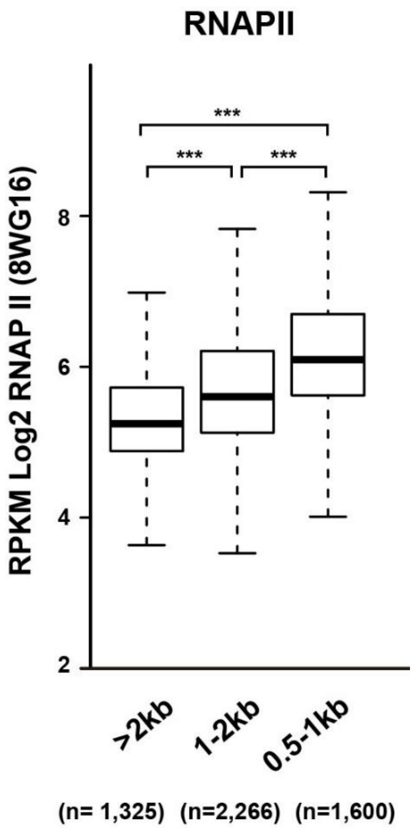

Supplementary Figure 5. The enrichment of RNAPII at the centers of transcribed regions is related to gene length

Boxplots for RNAPII (anti-8WG16; calculated as the $\log_2$ RPKM) at the centers of transcribed regions versus gene groups clustered by gene length cluster (i.e., >2 kb, 1-2 kb, and 0.5-1 kb). Asterisks (***) indicates p-value <0.001 calculated by Wilcoxon-and-Mann-Whitney tests.

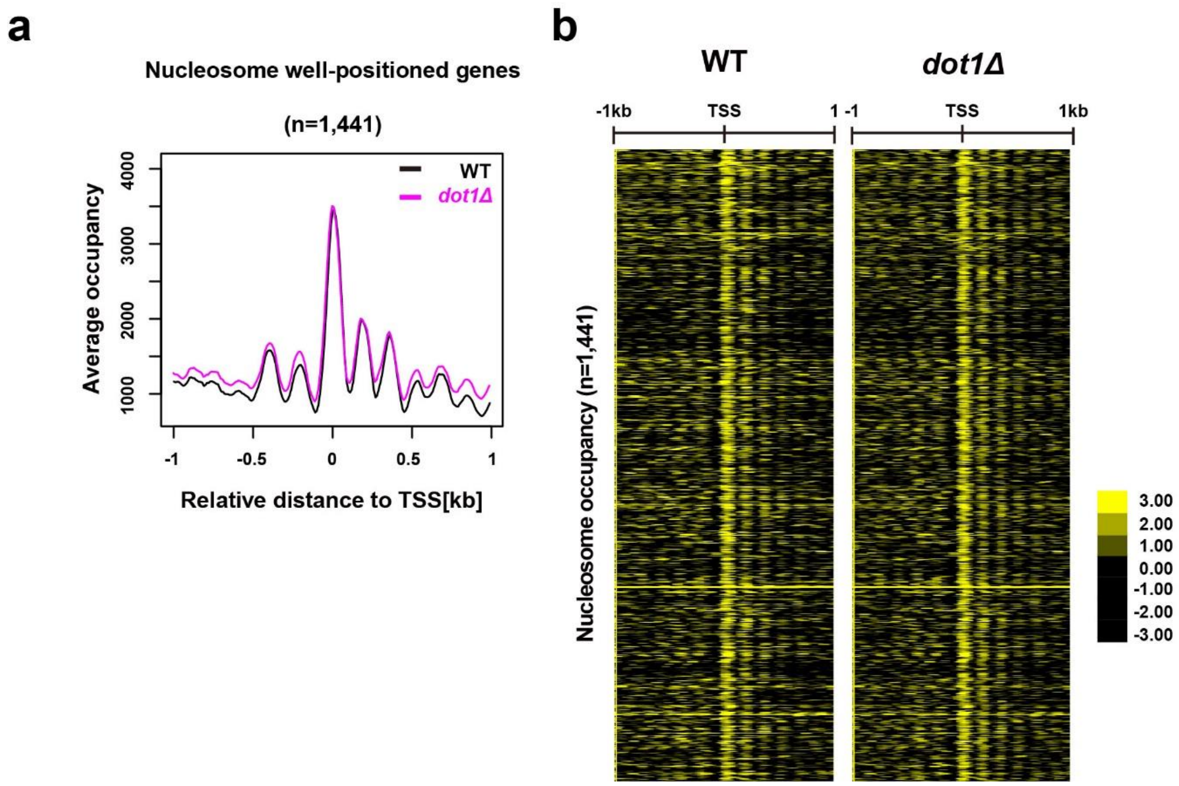

Supplementary Figure 6. Deletion of DOT1 does not clearly affect nucleosome positioning or occupancy

(a) Average plot of MNase-seq data, as analyzed by DANPOS2. Gray line indicates the average plot in wild-type cells, while the red line indicates the average plot in *dot1Δ* cells. The plot presents the 1 kb regions upstream and downstream of the TSSs of genes with well-positioned nucleosomes (n=1,441).

(b) Heatmap of MNase-seq data. Nucleosome occupancy was calculated as the $\log_{400}$ ratio of normalized values, using DANPOS2.

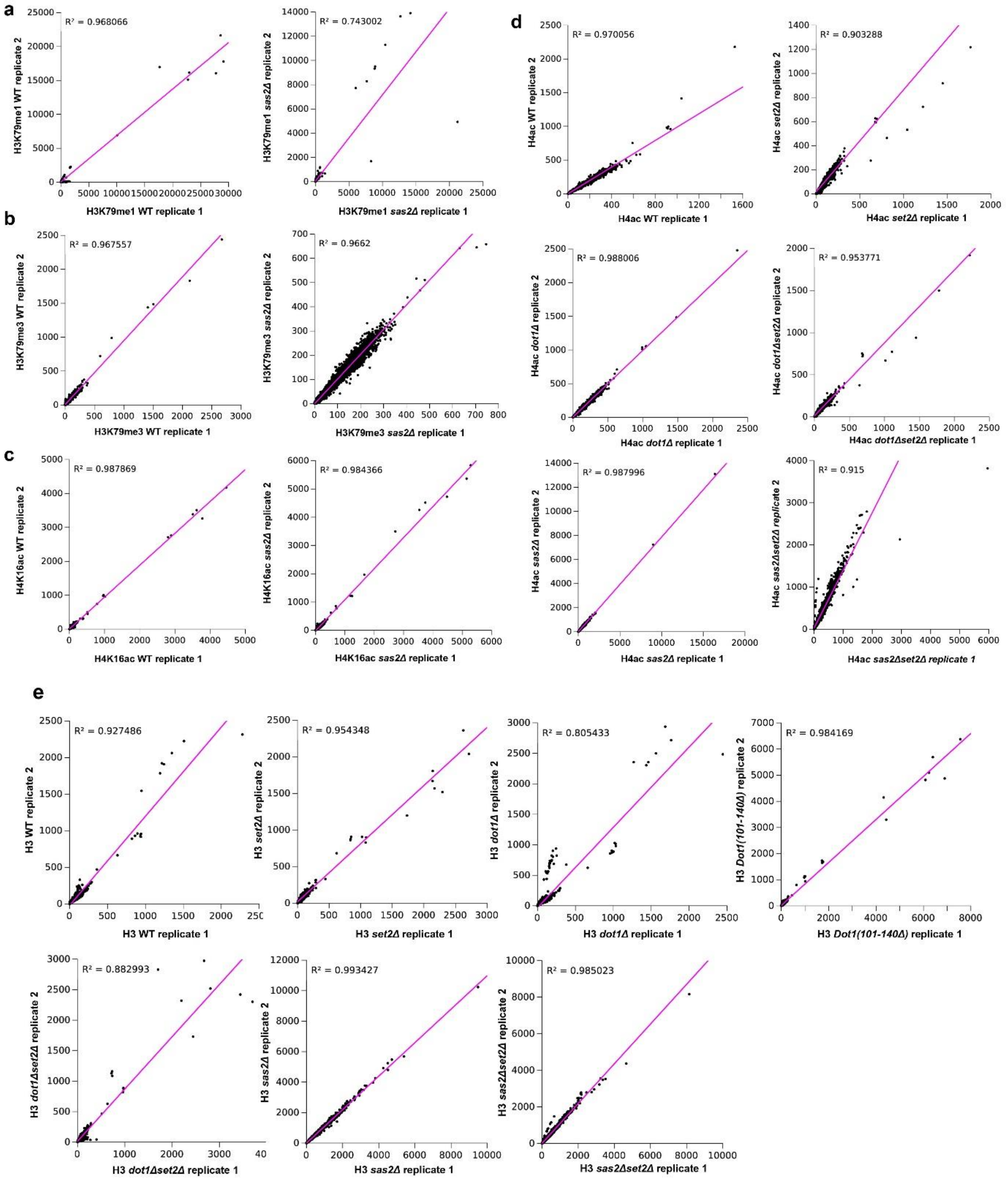

a
R² = 0.968066
H3K79me1 WT replicate 2
H3K79me1 WT replicate 1
R² = 0.743002
H3K79me1 sas2Δ replicate 2
H3K79me1 sas2Δ replicate 1
b
R² = 0.967557
H3K79me3 WT replicate 2
H3K79me3 WT replicate 1
R² = 0.9662
H3K79me3 sas2Δ replicate 2
H3K79me3 sas2Δ replicate 1
c
R² = 0.987869
H4K16ac WT replicate 2
H4K16ac WT replicate 1
R² = 0.984366
H4K16ac sas2Δ replicate 2
H4K16ac sas2Δ replicate 1
d
R² = 0.970056
H4ac WT replicate 2
H4ac WT replicate 1
R² = 0.903288
H4ac set2Δ replicate 2
H4ac set2Δ replicate 1
R² = 0.988006
H4ac dot1Δ replicate 2
H4ac dot1Δ replicate 1
R² = 0.953771
H4ac dot1Δset2Δ replicate 2
H4ac dot1Δset2Δ replicate 1
R² = 0.987996
H4ac sas2Δ replicate 2
H4ac sas2Δ replicate 1
R² = 0.915
H4ac sas2Δset2Δ replicate 2
H4ac sas2Δset2Δ replicate 1
e
R² = 0.927486
H3 WT replicate 2
H3 WT replicate 1
R² = 0.954348
H3 set2Δ replicate 2
H3 set2Δ replicate 1
R² = 0.805433
H3 dot1Δ replicate 2
H3 dot1Δ replicate 1
R² = 0.984169
H3 Dot1(101-140Δ) replicate 2
H3 Dot1(101-140Δ) replicate 1
R² = 0.882993
H3 dot1Δset2Δ replicate 2
H3 dot1Δset2Δ replicate 1
R² = 0.993427
H3 sas2Δ replicate 2
H3 sas2Δ replicate 1
R² = 0.985023
H3 sas2Δset2Δ replicate 2
H3 sas2Δset2Δ replicate 1

Supplementary Figure 7. ChIP-seq peak are highly reproducible

Pairwise correlations of ChIP-seq replicate signal at (a) H3K79 monomethylation (H3K79me1), (b) H3K79 trimethylation (H3K79me3), (c) H4K16 acetylation (H4K16ac), (d) H4 pan-acetylation (H4ac), and (e) H3 peaks in mutants and wild-type cells for whole number of genes (n=6,692). All data were obtained from biological duplicates. The RPKM in transcribed regions was taken as the peak occupancy. The correlation coefficient R^2 for each pairwise comparison is shown.

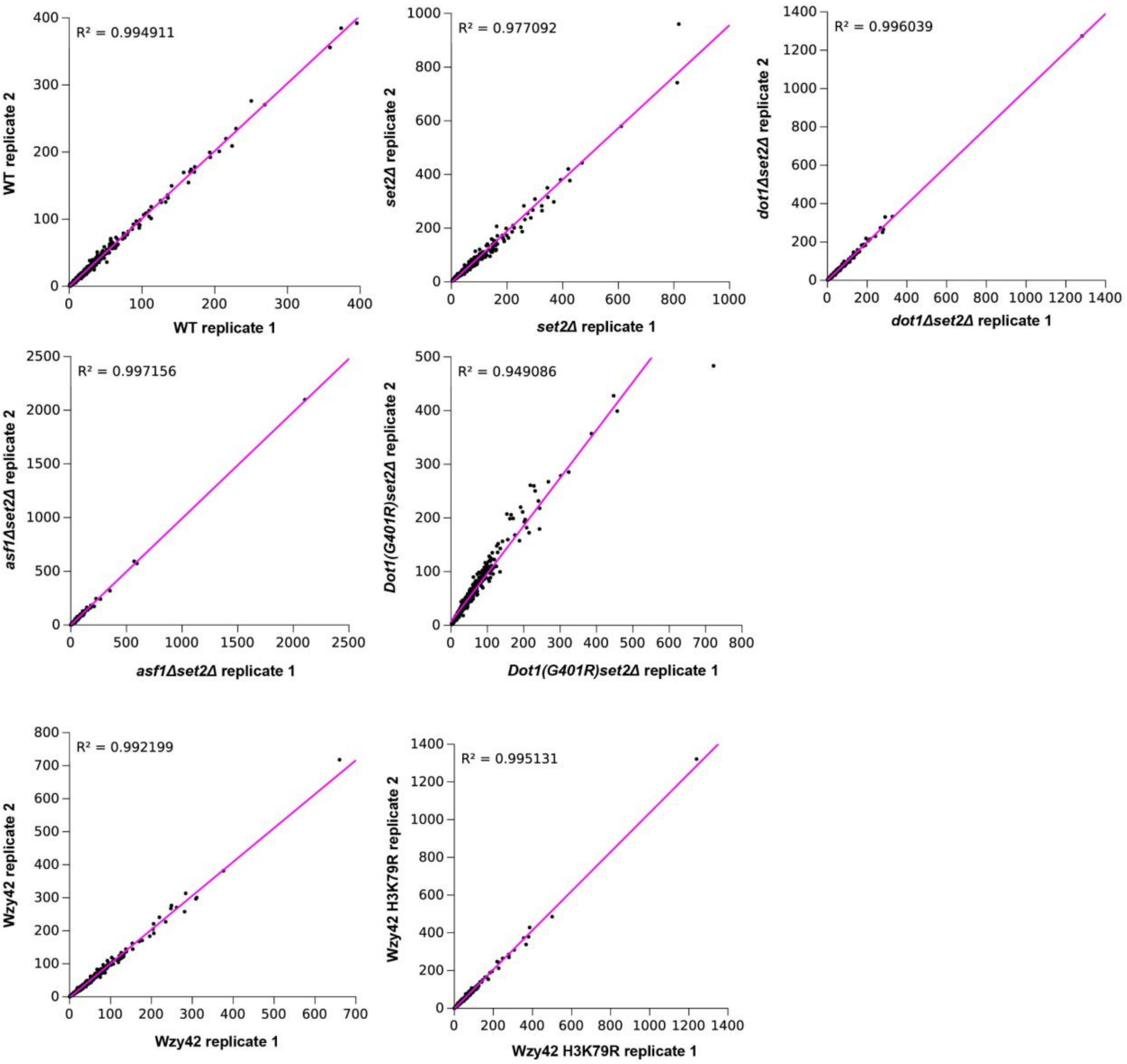

Supplementary Figure 8. mRNA-seq values are highly reproducible

Pairwise correlations of mRNA-seq replicate signal in mutants and wild-type cells for cryptic transcription genes (n=553). All data were obtained from biological duplicate samples. The RPKM in transcribed regions was taken as the peak occupancy. The correlation coefficient R^2 for each pairwise comparison is shown.

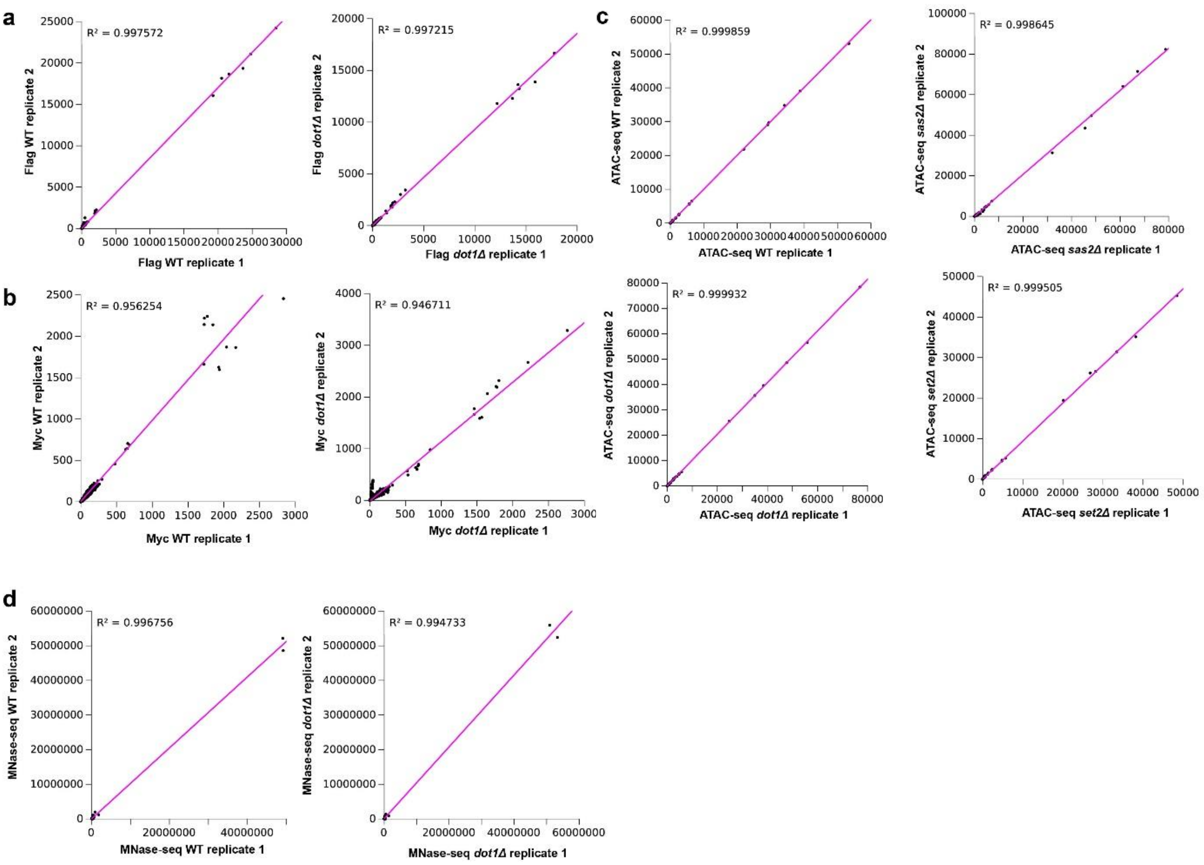

Supplementary Figure 9. ChIP-seq, ATAC-seq, and MNase-seq peak are highly reproducible

Pairwise correlations of ChIP-seq replicate signal at (a) Flag and (b) Myc in *dot1Δ* and wild-type cells for whole number of genes (n=6,692). (c) Pairwise correlations of ATAC-seq replicate signal in *sas2Δ*, *set2Δ*, *dot1Δ*, and wild-type cells for all genes (n=6,692). (d) Pairwise correlations of MNase-seq replicate signal in *dot1Δ* and wild-type cells for whole number of genes (n=6,692) All data were obtained from biological duplicate samples. (a-c) The RPKM in transcribed regions was taken as the peak occupancy for ChIP-seq and ATAC-seq. (d) The sum of calculated nucleosome occupancy value from DANPOS2 was taken as the peak occupancy for MNase-seq. The correlation coefficient R^2 for each pairwise comparison is reported.

a

b

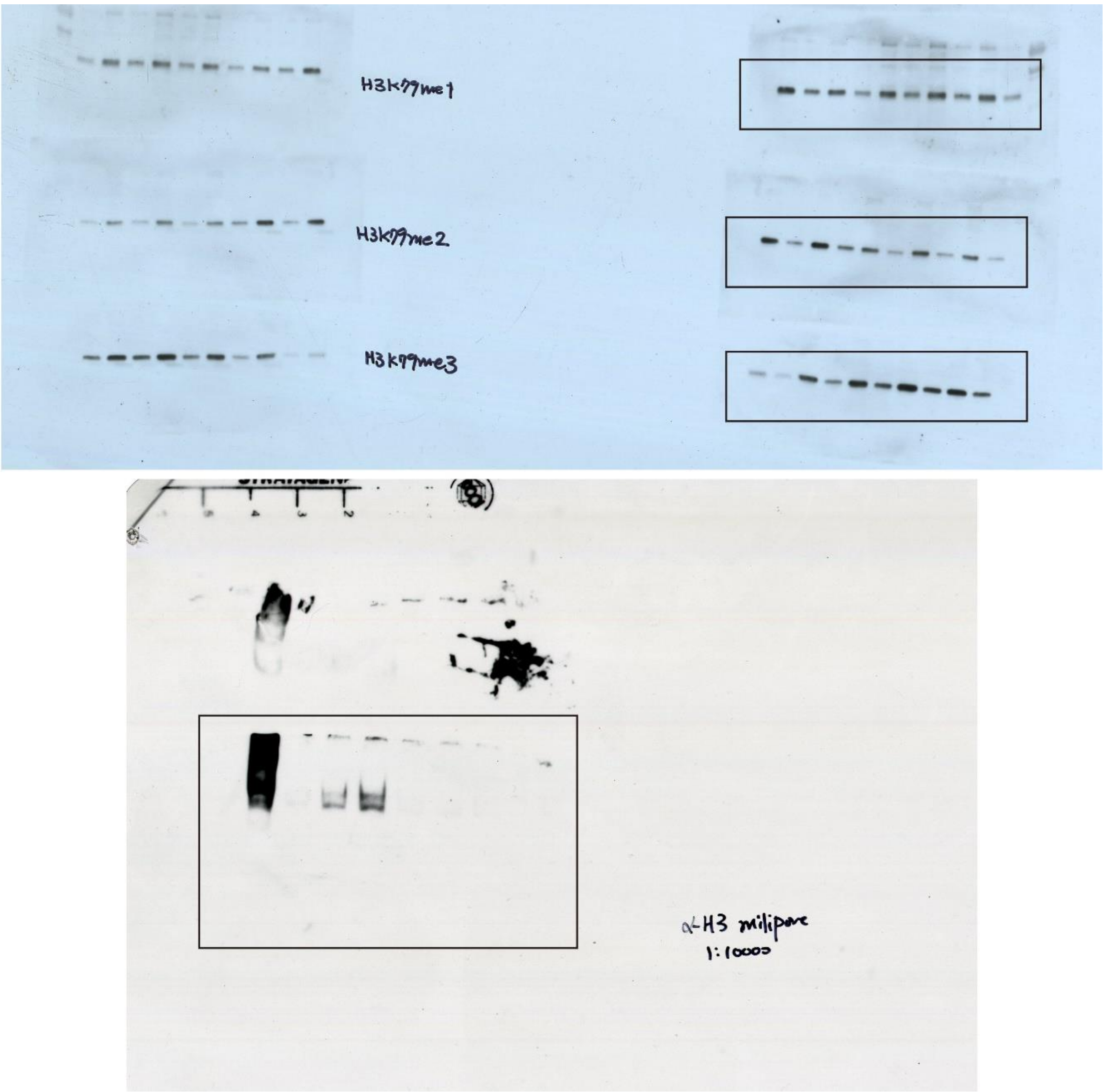

Supplementary Figure 10. Original Western blot film in Fig. 1 and Fig. 4

The uncropped scans of Western blot exposed to x-ray films in (a) Fig. 1a (b) Fig. 4c.

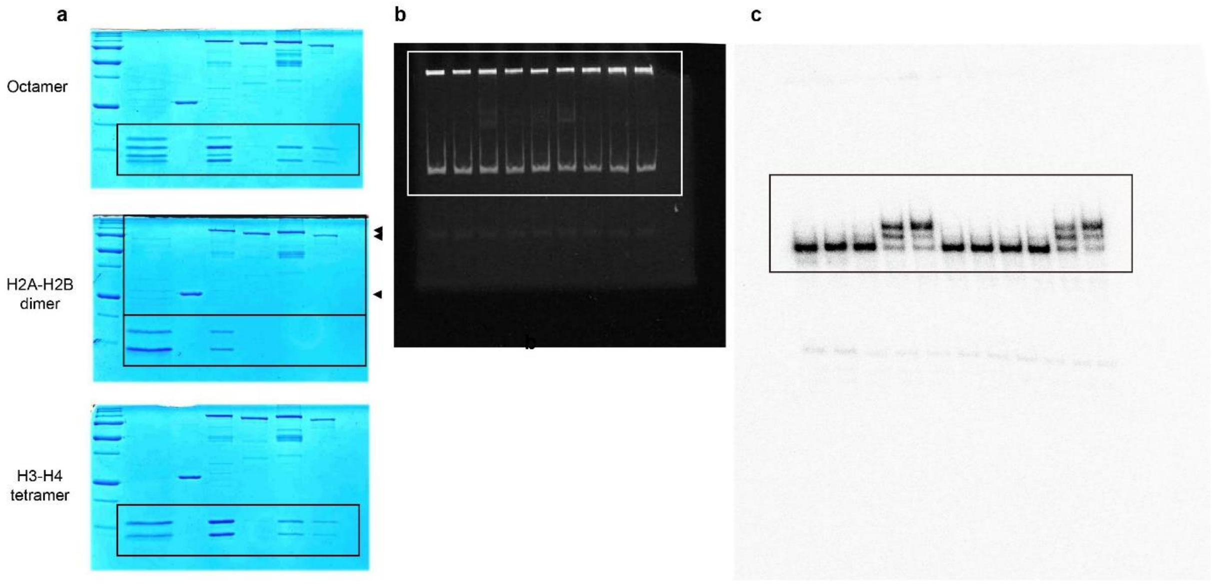

Supplementary Figure 11. Original blots images in Fig. 4

(a) The uncropped scan of coomasie blue stained SDS-PAGE gel in Fig. 4a. (b) The uncropped and pre-inversion image of EtBr stained native polyacrylamide gel in Fig. 4c. (c) The uncropped image of native polyacrylamide gel exposed using a FLA-7000 (Fuji) in Fig. 4d.

Supplementary Table 1. List of strains used in the study

Strain	Genotype	Parental strain	Source
wzy42	MATa; ura3−52 lys2−801 ade2−101 trp1Δ63 his3Δ200 leu2Δ1 hht1-hhf1::pWZ405-F2F9-LEU2 hht2-hhf2::pWZ403-F4F10-HIS3 ycP50-copyII(HHT2-HHF2-URA3)	wzy42	Roth, SY
ySY501	pWZ414-TRP1 HHT2-HHF2(K5A)	wzy42	This study
ySY503	pWZ414-TRP1 HHT2-HHF2(K8A)	wzy42	This study
ySY505	pWZ414-TRP1 HHT2-HHF2(K12A)	wzy42	This study
ySY507	pWZ414-TRP1 HHT2-HHF2(K16A)	wzy42	This study
SC007	pWZ414-TRP1 HHT2-HHF2(K16R)	wzy42	This study
SC008	pWZ414-TRP1 HHT2-HHF2(K12R)	wzy42	This study
w303a	MATa leu2-3,112 trp1-1 can1-100 ura3-1 ade2-1 his3-11,15	w303a	
ySH159	set2Δ::TRP1	w303a	This study
ySH160	dot1Δ::TRP1	w303a	This study
LPY4774	esa1-414	w303a	Pillus, L
ySY103	sas2Δ::KAN	w303a	This study
yHH302	LEU::pGAL1-FLO8-HIS3	w303a	This study
yHH302	LEU::pGAL1-FLO8-HIS3 set2Δ::TRP	w303a	This study
yHH302	LEU::pGAL1-FLO8-HIS3 sas2Δ::HYGMX set2Δ::TRP	w303a	This study
yHH302	LEU::pGAL1-FLO8-HIS3 dot1Δ::HYGMX set2Δ::TRP	w303a	This study
ySH1020	set2Δ::HIS3 dot1Δ::TRP1	w303a	This study
ySH1022	set2Δ::HIS3 Dot1(G401R)	w303a	This study
ySH1024	set2Δ::HIS3 Dot1(101-140Δ)	w303a	This study
yHH532	set2Δ::NAT	wzy42	This study
SC546	set2Δ::HYG H3K79R	wzy42	This study
SC542	asf1Δ::NAT set2Δ::HYG	w303a	This study
SC528	asf1Δ::KAN set2Δ::HYG dot1Δ::TRP1	w303a	This study
ySH1026	bar1Δ::KAN	w303a	This study
SC526	bar1Δ::KAN sas2Δ::HYG	W303a	This study
ySH1028	bar1Δ::KAN dot1Δ::TRP1	w303a	This study
ySH1055	bar1Δ::NAT Dot1(101-140Δ)	w303a	This study

SC544	bar1Δ::HYG set2Δ::TRP1	w303a	This study
SC514	bar1Δ::HYG set2Δ::HIS3 dot1Δ::TRP1	w303a	This study
SC667	bar1Δ::HYG set2Δ::TRP1 sas2Δ::HYG	w303a	This study
YKW409	MATa; lys2−801 ade2−101 trp1Δ63 his3Δ200 leu2Δ1 hht1-hhf1::pWZ405-F2F9-LEU2 hht2-hhf2::pWZ403-F4F10-HIS3 [pNOY439(CEN6 ARS4-TRP1 MYC-HHT2-HHF2)] ura3-52: YIplac211pGAL1/10-FLAG-HHT1-HHF1 bar1Δ::KAN<br>bar1Δ::KAN	wzy42	This study
YKW410	as YKW409, dot1Δ::NAT	wzy42	This study

Supplementary Table 2. List of antibodies used in the study

Antibody target	Supplier	Dilution	Catalog#
Flag	Sigma	1:200 (for ChIP)	F1804
Myc	prepared by Lee Lab	1:100 (for ChIP)	
Histone H3	prepared by Lee Lab	1:1000; 1:200 (for ChIP)	
Histone H3	Millipore	1:10000	05-928
Pan-acetylated H4	Millipore	1:200 (for ChIP)	06-866
H3K79me3	prepared by Lee Lab	1:2000; 1:200 (for ChIP)	
H3K79me2	prepared by Lee Lab	1:1000	
H3K79me1	prepared by Lee Lab	1:1000; 1:100 (for ChIP)	
H4K16ac	prepared by Lee Lab	1:1000; 1:200 (for ChIP)	
Dot1	prepared by Lee Lab	1:1000	
RNAPII 8WG16	Biolegend Inc.	1:50 (for ChIP)	mms-126R

Supplementary Table 3. List of primers used in the study

Gene	Sequence
PCA1#1_F	CAAAGGTTATAGGTGCCAGAG
PCA1#1_R	GCAGCCTACACGAACTAAAT
PCA1#2_F	CAGATGCTGTCTCCACTATTAAC
PCA1#2_R	CGTTGCGTGAGAACGAATA
FLO8#1_F	CAATTCCRCCCACGGAACAACCGTAC
FLO8#1_R	CCGCCGTAGGTTGATTTATCCACTGCTG
FLO8#2_F	GAGATCGTAATCCGGTCCTTGGTCTC
FLO8#2_R	GAAACCTCTGCATCTACAAATAGTGGCG
Actin_F	GTGTGATGTCGATGTCCGTAAG
Actin_R	CGGAGTACTTTCTTTCTGGAGG
Lateral_F	GGATCCTCTAGAGTCGGGAGC
Lateral_R	CTGGAGAATCCCGGTGCC
Central_F	CTGGCACCGGCAAGGTCG
Central_R	TCCCTTATGTGATGGACC
